# Real-Time Demonstration of Split Skin Graft Inosculation and Integra Dermal Matrix Neovascularization Using Confocal Laser Scanning Microscopy

**Published:** 2009-08-20

**Authors:** John Greenwood, Mahyar Amjadi, Bronwyn Dearman, Ian Mackie

**Affiliations:** Burns Unit, Royal Adelaide Hospital, North Terrace Adelaide, SA 5000, Australia

## Abstract

**Objectives:** During the first 48 hours after placement, an autograft “drinks” nutrients and dissolved oxygen from fluid exuding from the underlying recipient bed (“plasmatic imbibition”). The theory of inosculation (that skin grafts subsequently obtain nourishment via blood vessel “anastomosis” between new vessels invading from the wound bed and existing graft vessels) was hotly debated from the late 19th to mid-20th century. This study aimed to noninvasively observe blood flow in split skin grafts and Integra™ dermal regeneration matrix to provide further proof of inosculation and to contrast the structure of vascularization in both materials, reflecting mechanism. **Methods:** Observations were made both clinically and using confocal microscopy on normal skin, split skin graft, and Integra™. The VivaScope™ allows noninvasive, real-time, in vivo images of tissue to be obtained. **Results:** Observations of blood flow and tissue architecture in autologous skin graft and Integra™ suggest that 2 very different processes are occurring in the establishment of circulation in each case. Inosculation provides rapid circulatory return to skin grafts whereas slower neovascularization creates an unusual initial Integra™ circulation. **Conclusions:** The advent of confocal laser microscopy like the VivaScope 1500™, together with “virtual” journals such as *ePlasty*, enables us to provide exciting images and distribute them widely to a “reading” audience. The development of the early Integra™ vasculature by neovascularization results in a large-vessel, high-volume, rapid flow circulation contrasting markedly from the inosculatory process in skin grafts and the capillary circulation in normal skin and merits further (planned) investigation.

As plastic surgeons, the skin is our organ of practice and the knowledge of how skin grafts take is one of the fundaments of our art. Our trainers and our textbooks tell us that there are 2 distinct, sequential processes. During the first 48 hours after graft placement, the graft “drinks” nutrients and dissolved oxygen from fluid exuding from the underlying recipient bed. This process became known as “plasmatic imbibition,” a term first coined by Hübscher^1^ in 1888. At that time (and for the next 70 years), there was considerable debate as to what came next. Bert^2^ used “abouchement” in 1865 to describe the process whereby vessels budding from the wound surface connected to existing dermal vessels in the graft, like mouths meeting. Thiersch^3^ preferred the term “inosculation,” derived from the Latin *inosculare*, to kiss. However, an alternative movement, centered on Garré^4^ and Hübscher,^1^ believed that new vessel in growth from the bed into the graft had to occur because the graft vessels were irreversibly occluded after harvesting. The debate continued into the 20th century, fueled in 1925 by the finding by Davis and Traut^5^ of anastomoses between bed and graft vessels by 22 hours in dogs. In 1952, Conway and colleagues^6^ and Ham^7^ swung the pendulum the other way again, with independent studies addressing varying animal models and differing techniques, concluding that vascularization of grafts did not occur. The following year, Taylor and Lehrfeld,^8^ using direct stereomicroscopy, visualized graft vascularization and were supported by Scothorne and McGregor^9^ in the same year. In March 1956, Converse and Rapaport^10^ again used stereomicroscopy to visualize blood cells traveling in vessels within the graft in vivo within 3 or 4 days of graft application.

The problem with such definitive evidence lay in the inability of researchers to demonstrate it to the reading audience because film, or even video evidence, could not be submitted to paper journals. Only now, with the advent of e-Journals, can such evidence be mass visualized. Here, we report the results of observations made with a confocal laser scanning microscope (CLSM), the Vivascope 1500 (Lucid, Inc, Rochester, NY).

The Vivascope 1500 is described as an optical biopsy system. Its confocal laser scanning configuration allows serial real-time visualization in horizontal “sections” (of chosen thickness) from the surface of the keratin layer, through the epidermis, and into the superficial reticular dermis. Offering noninvasive, high-resolution views of skin, its primary use has been for dermatological applications. It has the potential to allow skin assessment, which may eventually abolish the need for invasive skin biopsies and histological processing (which has deleterious effects on tissue biopsy structure, including dehydration, morphological change, and artifact creation).

Although the microscope has been mainly used for diagnostic imaging of skin tumors and other skin disorders, its research-related applications are increasing. Recent publications by Altintas et al^11^ have followed experience with confocal laser scanning microscope to evaluate and differentiate burn depth. However, only superficial and deep partial-thickness burns have (as yet) been assessed. Using the CLSM for analyzing split-thickness graft and Integra in full-thickness burns is novel, with never-before published images.

We report the results of observations of in vivo real-time visualization of blood flow within vessels of a split skin graft 4 days after the placement of the graft. Additional observations of erythrocyte movement through larger (˜28-µm diameter) neovascular channels within Integra at 23 days are also presented.

## MATERIALS AND METHODS

### Patient

**Figure 1:**
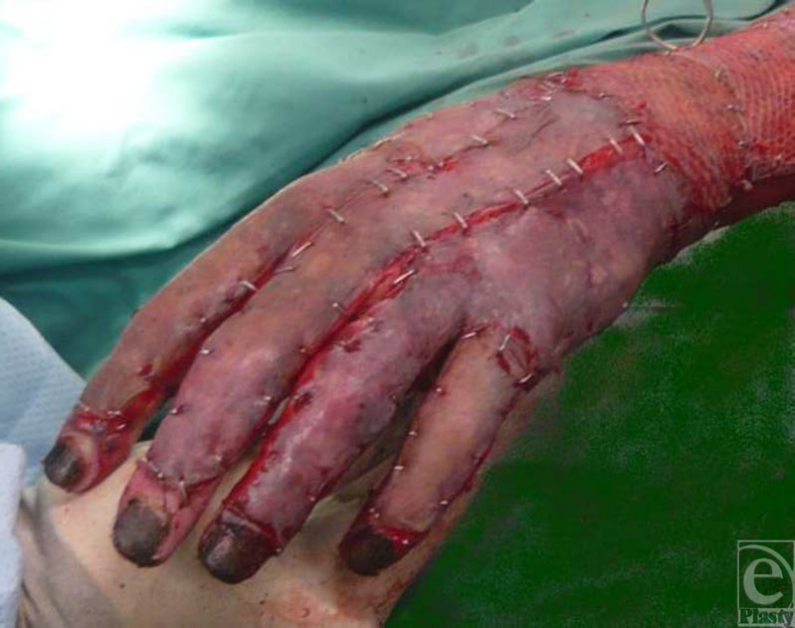
Thick sheet split-skin graft immediately after application to the left hand.

**Figure 2:**
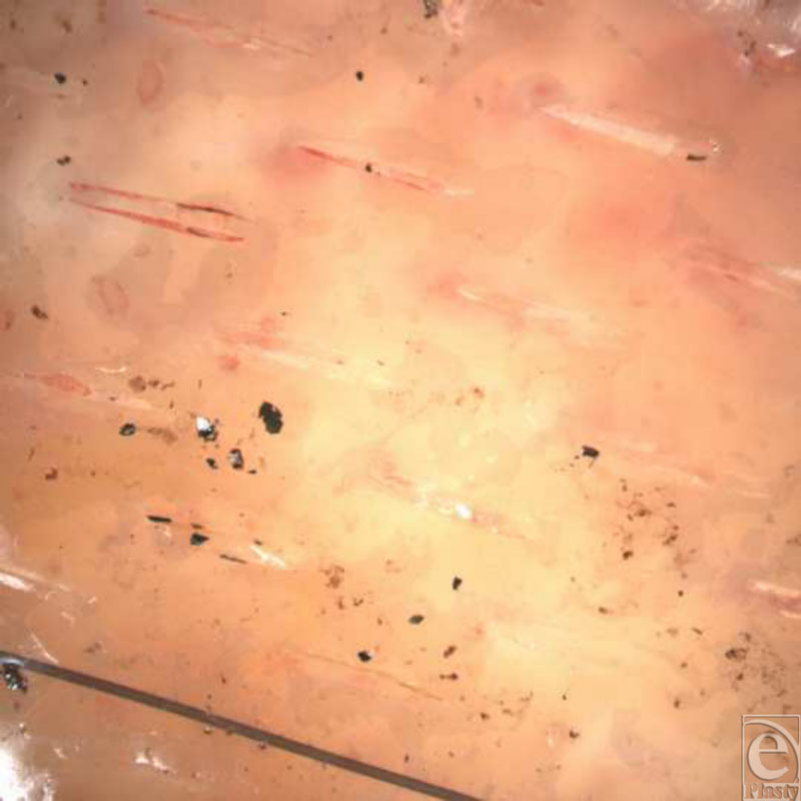
“Macro” image (VivaCam) of debrided chest burn on day 13 after the application of meshed Integra. Despite the color, no evidence of blood flow was found on Vivascopy.

**Figure 3:**
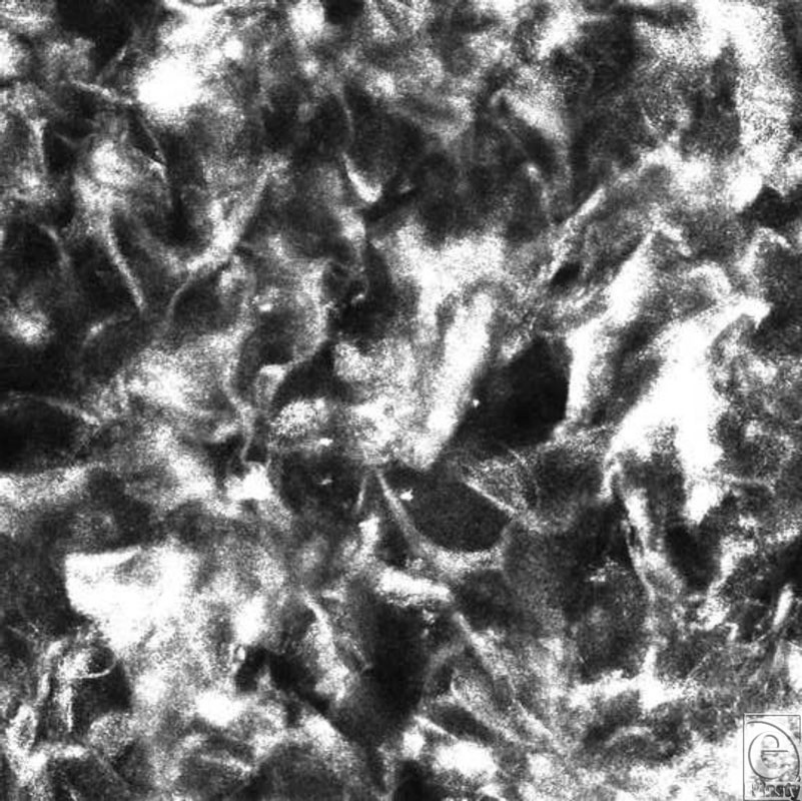
Vivascope still image of Integra at the time of application. The collagen network is visible and devoid of cellular components.

All observations were made in a 38-year-old man who presented with 80% total body surface area (TBSA) burn, 60% of which were full thickness. The treatment of such major burns varies slightly according to site. As is common practice, the hands were treated with thick split-thickness sheet autograft and it was such a graft to the dorsum of his left hand that features in our video observations (Fig 1). Day 4 was the earliest that the graft was deemed to be robust enough for “Vivascopy.” After the initial debridement, the remaining wounds were covered with Integra Dermal Regeneration Template (Figs 2 and 3). Multiple observations of the Integra during the first 3 weeks were made, with no evidence of any blood flow. Following observation on day 23, the silicone layer of Integra was delaminated and split skin graft was applied.

### Instrument

A CLSM (Vivascope 1500) was purchased from Lucid, Inc, Rochester, NY, for in vivo examinations on burn patients. This microscope is a noninvasive tool that illuminates a small volume of tissue from which reflected light is collected and projected onto a detector through a small pinhole. The pinhole rejects all backscattered light from focus planes, thus enabling high-resolution images. The Vivascope takes horizontal (enface) sections from the tissues epidermal surface to the superficial reticular dermis at the cellular level. It allows visualization of real-time biological processes, such as blood flow up to a depth of 200 to 250 µm. This is sufficient to image the epidermis and upper dermis (papillary dermis and upper reticular dermis) in normal skin. For detailed microscope specifications, see www.lucid-tech.com. Briefly, the Vivascope uses a near-infrared laser wavelength at 830 nm; it is equipped with a 30× water-immersion objective lens, with a numerical aperture of 0.9. Each image has a field of view of 500 × 500 µm. The user can image in three dimensions within living tissue by changing focus and/or movement of the tissue ring. A sequence of images can be captured to form a mosaic or block across a plane parallel to the skin surface of up to 8 × 8 mm. Similarly, a sequence of images can be captured in vertical steps to display a *z*-stack (Vivastack). Video at 15 to 25 frames per second can also be captured to document dynamic events such as blood flow. Resolution is similar to histology, with lateral resolution of approximately 1 µm and a vertical resolution of 3 to 5 µm. In reflectance confocal imaging, structures that have a high refractive index compared with the surrounding medium appear bright, such as melanin with a refractive index of 1.7.^12^ Both individual cells and cell patterns can assist with identification, orientation, and evaluation of tissue in real time.

### Imaging

A small drop of oil was applied to the area under investigation as an immersion medium between the adhesive plastic window and the skin. A metal tissue ring was gently placed over the area. The tissue ring has an adhesive component that was not exposed in the early stages because of graft fragility. The Vivacam digital dermoscopic camera was applied to the ring to capture macroscopic images at the surface level. Ultrasound gel was applied between the plastic window and the lens to avoid air interference. This enables tissue viewing because the refractive index of the gel (1.34) is close to that of the epidermis.^13^ Laser power is less than 30 mW and causes no tissue damage. The skin was scanned layer by layer, and with the use of VivaScan v7 software, automated Vivastacks (composed of still confocal images) and videos were captured.

As mentioned, several landmarks make orientation possible. One of the most notable of these landmarks is the rete peg, in which the epidermis appears to be “punctured” by a dermal “spike,” surrounded by a rim of basal cells. Because each rete peg is centered by a knot of dermal capillaries, it was these areas on which we concentrated our observations on the skin graft. Multiple observations were required within the Integra.

## RESULTS

### Graft

**Figure 4:**
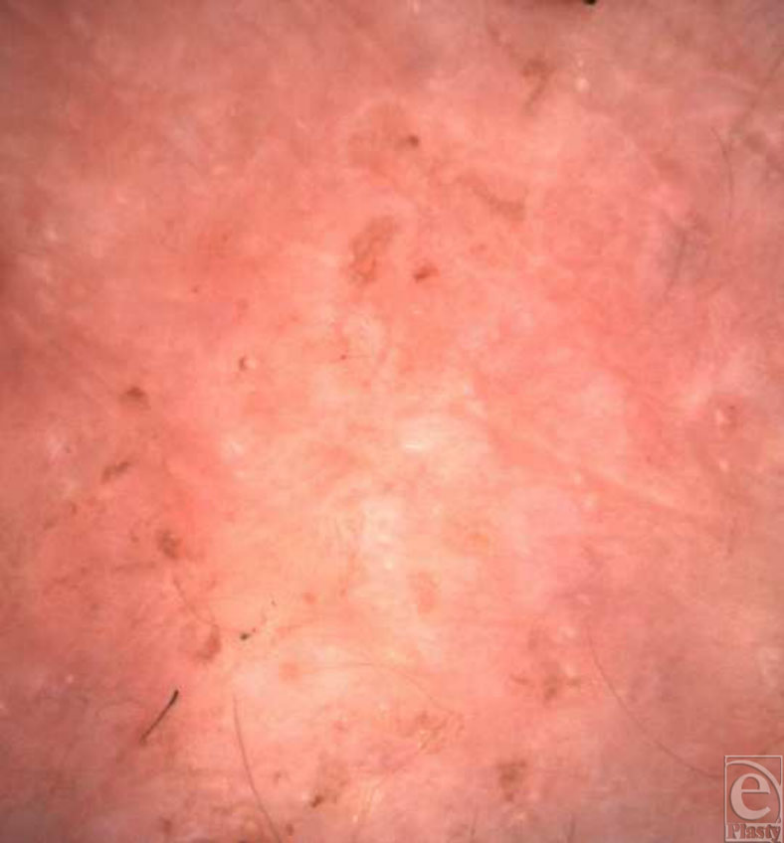
“Macro” image (VivaCam) of sheet skin graft left hand on day 4 after application.

On day 4 post-application, “macro” examination of the sheet graft on the left hand revealed a healthy pink coloration, whereas Vivascopic examination (Fig 4) of the graft revealed blood flow (erythrocyte movement) clearly visualized within the central vessels of the rete pegs of the grafted skin (Video 1).

**Figure:**
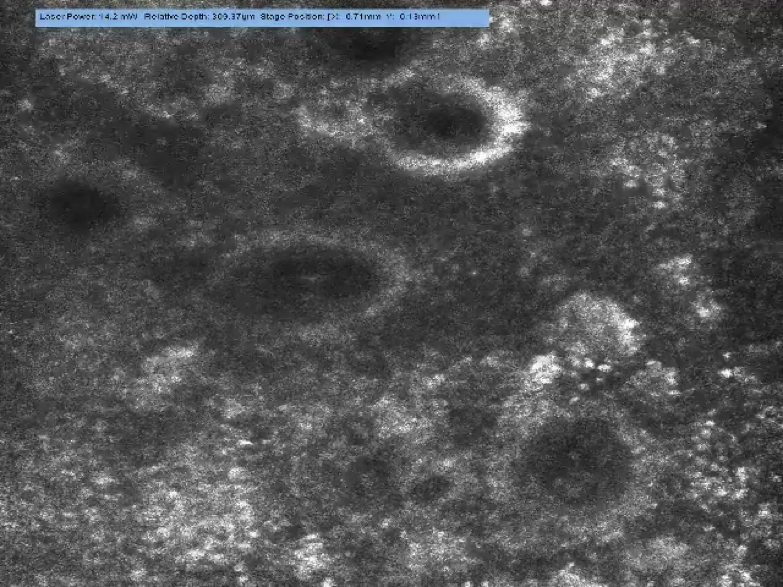
Video 1. Vivascope video image of blood flow in rete vessels on day 4 post-application of split skin graft showing “single file” slow movement of blood cells through capillaries.

**Figure:**
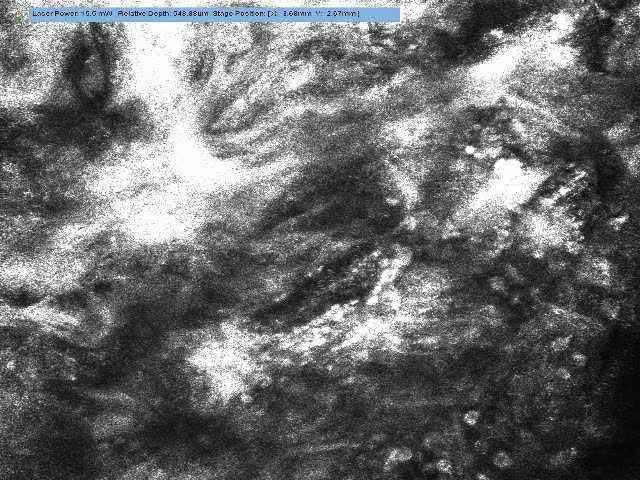
Video 2. Vivascope bifocal microscopy image taken on day 23 post-application of Integra, demonstrating larger neovascular channels containing a large volume of rapidly moving blood cells. The collagen matrix is populated by dermal cells.

It must be remembered that thick split skin grafts contain the entire epidermis and a portion of dermis (down to mid-dermal level in the thickest split skin grafts). The rete pegs are therefore graft structures, not recipient site structures. The flow can be visualized in real time and, because the graft was applied to the underlying fat, the feeding vessels can be neogenic vessels only from the subcutis. It is noticeable that the flow is comparable with normal skin capillaries because blood cells move through them in single file (Video 3).

**Figure:**
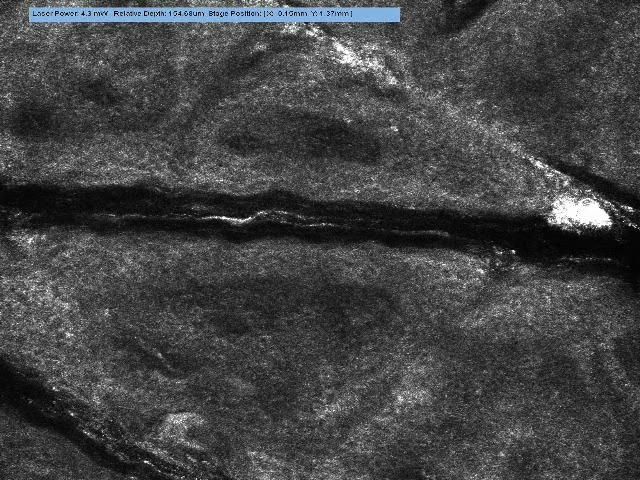
Video 3. Vivascope video image of capillary blood flow in normal skin showing similar “single file” slow flow of blood cells. Some movement vertically is demonstrated by the Vivascope, allowing vessels at different depths to be visualized.

### Integra

Clinically, a color change (white raw Integra to peachy or coral-colored vascularized Integra) is used to determine when Integra has been vascularized sufficiently to allow delamination and skin graft application, a change that is not usually apparent in the acute major burn wound until approximately day 14 post-application. Even then, delamination and grafting may be premature and the graft may struggle or fail. Baseline observation on Integra showed merely a porous lattice of collagen fibers (Fig 2). These fibers persist after fibrovascular invasion from the dermal bed, but there was no indication as to where a search for neovasculature should be concentrated. We began observations once the Integra appeared to be firmly adhered to the recipient bed (day 9 post-application). Despite evidence of autologous collagen being laid down and some localized evidence of blood cell movement on day 20, blood flow was not reliably recorded until day 23 when a series of large-diameter (˜28 µm) vessels was noted with rapid blood cell (erythrocyte 6–8 µm and leucocyte 12–15 µm) movement within (Video 2). The flow was very fast through these large vessels and was not constrained to a single-file stream in contrast to the flow through the visualized graft and normal skin capillaries.

## DISCUSSION

When one considers the speed with which split skin grafts “pink up” and the relative latency of neovascularization; inosculation has always made the most sense as the mechanism for the reestablishment of blood flow within grafts. To date, however, it has not been possible for journal readers to visualize direct observations of such phenomenon in vivo. In addition, as far as it can be ascertained from literature searches, no real-time demonstration of the vascularization of Integra either in vivo or in vitro has ever been demonstrated. Neovascularization should be expected to (and does) take much longer than inosculation since the angiogenetic process alone is responsible for a neovasculature to invade and supply a relatively thick structure. For example, the vascularization of Integra dermal matrix takes approximately 2 weeks in the acute (hot) burn setting but can take 4 to 5 weeks in reconstructive (cold) cases. This vascularization can occur only from the wound bed since no vascular elements exist in Integra. In fact, the tardiness exhibited by Integra during neovascularization should act as additional proof of inosculation in skin grafts (if any were necessary).

Stern and colleagues reported a histological analysis of Integra from 10 US centers but only briefly mentioned that “new vascularization was present with sprouting endothelial cells.”^14^ In 2006, Moiemen and colleagues^15^ described Integra “take” as occurring in four stages: imbibition, fibroblast invasion, neovascularization, and remodeling/maturation. Using histological staining techniques, they observed that neovascularization began in the second week with migration of endothelial cells. These cells formed solid structures that stained positively for the endothelial markers CD31 and CD34. Not until the third week did they observe lumen formation and full establishment of the neovasculature by the end of the fourth week. They fail to describe the size of the vessels and, of course, could not visualize blood flow. Their timings, however, correlate exactly with the clinical course described earlier (in keeping with our own experience).

The initial steps of vascularization are likely to follow the upregulation of bone-marrow–derived progenitor cells that enter the under surface of the Integra in the wound bed exudate. Differentiation and subsequent signaling will ensure the process of vessel formation continues. Why the early neovasculature in Integra should be of such large diameter (˜28 µm) needs further evaluation. It may be that the structural constraints of an Integra dermis do not limit expansion, or since inhibition signaling is necessary for regulation of vessel growth that the lack of any vascular element within Integra allows unrestrained early development. It may simply be that what we are observing is some sort of “proto-vessel” development that does not differentiate into a recognizable vasculature but remains as rudimentary vascular loops, providing buds for inosculation with an overlying skin graft. The vasculature observed throughout the Integra can be compared with previous findings in malignant melanoma, squamous cell carcinoma, and inflammatory diseases such as Psoriasis.^16^ The irregular orientation is similar to that seen with melanoma and SSC, whereas the enlarged vessels are similar to that seen with dermatitis.^17^

It would be interesting to visualize these same vessels later, to observe whether maturation results in development of a recognizable “capillary” structure, although the application of the skin graft over vascularized Integra to afford definitive closure might make such visualization impossible.

Future work that hopes to answer some of the questions raised would traditionally rely on tissue biopsying or other invasive procedures; which somewhat negates the noninvasiveness of the VivaScope observations. We are investigating the potential of fluorophores that can be used to label human cells in vivo and be visualized by different laser wavelengths provided by Lucid Inc. A number of collaborations are developing within SA Pathology with cell signaling and vascular biology groups to continue this work.

## CONCLUSIONS

The development of e-Journals has brought hitherto impossible data to the “reader.” Inosculation can be the only explanation for the rapid reestablishment of blood flow to split skin grafts. Integra revascularization is by neo-angiogenesis, takes longer, and is characterized (at least initially) by more rapid, higher-volume flow through larger vessels. Further collaborative work is planned to investigate this phenomenon. Real-time in vivo confocal laser microscopy could be a valuable tool in wound healing research.
